# Predicting Major Preoperative Risk Factors for Retears After Arthroscopic Rotator Cuff Repair Using Machine Learning Algorithms

**DOI:** 10.3390/jcm14061843

**Published:** 2025-03-09

**Authors:** Sung-Hyun Cho, Yang-Soo Kim

**Affiliations:** Department of Orthopedic Surgery, Seoul St. Mary’s Hospital, The Catholic University of Korea, Banpo-Daero 222, Secho-gu, Seoul 06591, Republic of Korea; piach197@gmail.com

**Keywords:** arthroscopic rotator cuff repair, machine learning, retear

## Abstract

**Background/Objectives**: This study aimed to identify the risk factors for retears after arthroscopic rotator cuff repair (ARCR) and to establish a hierarchy of their importance using machine learning. **Methods**: This study analyzed 788 primary ARCR cases performed by a single senior surgeon from January 2016 to December 2022. The condition of the repaired supraspinatus was assessed via magnetic resonance imaging (MRI) or sonography within 2 years after surgery. In total, 27 preoperative demographic, objective, and subjective clinical variables were analyzed using five well-established models: Extreme Gradient Boosting (XGBoost), Random Forest (RF), Support Vector Machine (SVM), Neural Network (NN), and logistic regression (LR). The models were trained on an 8:2 split training and test set, with three-fold validation. The primary metric for evaluating model performance was the area under the receiver operating characteristic curve (AUC). The top five influential features were extracted from the best-performing models. Univariate and multivariate LRs were performed independently as a reference. **Results**: The overall retear rate was 11.9%. The two best-performing prediction models were RF (validation AUC = 0.9790) and XGBoost (validation AUC = 0.9785). Both models consistently identified the tear size in the medial–lateral (ML) and anterior–posterior (AP) dimensions, full-thickness tears, and BMI among the top five risk factors. XGBoost uniquely included female sex, while RF highlighted the visual analogue scale (VAS) pain score. While conventional univariate regression indicated multiple significant factors associated with retears (age, full-thickness tear, AP and ML tear size, biceps conditions, fatty infiltration of three rotator cuff muscles, and atrophy of supraspinatus), multivariate analysis demonstrated that only age and the ML tear size are significant factors. **Conclusions**: Machine learning models demonstrated enhanced predictive accuracy compared to traditional LR in predicting retears, and the importance of risk factors was derived. Tear size, full-thickness tears, BMI, female sex, and VAS pain score emerged as the most influential risk factors.

## 1. Introduction

Arthroscopic rotator cuff repair (ARCR) is a well-established treatment for rotator cuff tears. Despite its effectiveness, the occurrence of tendon retears post repair remains a significant clinical challenge. Numerous studies have sought to identify and predict the risk factors for retears following ARCR [1,2,3,4,5,6,7]. The reported risk factors vary and include age, tear size, critical shoulder angle, and fatty infiltration (FI) of the tendon. However, findings regarding these factors have been inconsistent, with some studies reporting contradictory outcomes [3,6,7]. This indicates that the issue is complex and warrants further exploration.

The majority of research on retear risk factors after ARCR has focused on objective measures, often relegating subjective factors to the margins. For example, Kwon et al. [3] proposed a scoring system to assess rotator cuff healing post ARCR that included six objective factors but did not incorporate patient-reported outcome measurements (PROMs). This reflects a broader trend in research that prioritizes objective indicators over subjective ones, such as the visual analogue scale (VAS) pain score or the American Shoulder and Elbow Surgeon (ASES) score. While objective factors are undoubtedly important, the dismissal of subjective factors overlooks their potential significance. Numerous studies have identified a significant correlation between a patient’s subjective pain experiences and adverse prognosis, suggesting that integrating subjective factors with objective metrics could provide a more comprehensive understanding of retear risk factors [8,9,10].

Alongside these clinical investigations, the introduction of machine learning techniques has presented new methodologies for analyzing retear risk factors [1,11,12,13]. Harada et al. [1] used a decision tree algorithm to analyze data from 286 patients. Although this sample size may seem small for machine learning applications, the study demonstrated the potential of machine learning in predicting retears. Subsequent research has not only leveraged machine learning algorithms for prediction but also for identifying and prioritizing risk factors, using tools such as SHapley Additive exPlanation (SHAP) values and models such as Extreme Gradient Boosting (XGBoost) and Random Forest (RF), which have shown high predictive accuracies [11,14,15,16]. These efforts have been documented across multiple publications [11,17,18]. However, this innovative approach to exploring risk factors has yet to be applied specifically to retears after ARCR.

Therefore, this study aims to identify the risk factors influencing retears following ARCR using multiple machine learning algorithms and to rank and reveal the relative importance of these risk factors using high-accuracy machine learning models. Our approach includes both objective parameters and PROMs, providing a holistic view of predictive factors for retears. We hypothesize that (1) machine learning algorithms will not only predict retears after ARCR with high precision but also facilitate a ranking of risk factors and (2) that, while objective factors will emerge as significant predictors, some subjective factors should also be considered important risk factors.

## 2. Materials and Methods

Study approval was obtained from the institutional review board of our hospital. A total of 1042 adult patients underwent ARCR at our hospital from January 2016 to December 2022. Included in this study were patients who had full- or partial-thickness tears of the supraspinatus (SSP) tendon with or without combined tears of other rotator cuff tendons and underwent arthroscopic repair, a minimum follow-up period of 1 year, and radiological records available to evaluate the integrity of the repaired tendon.

**Figure 1:**
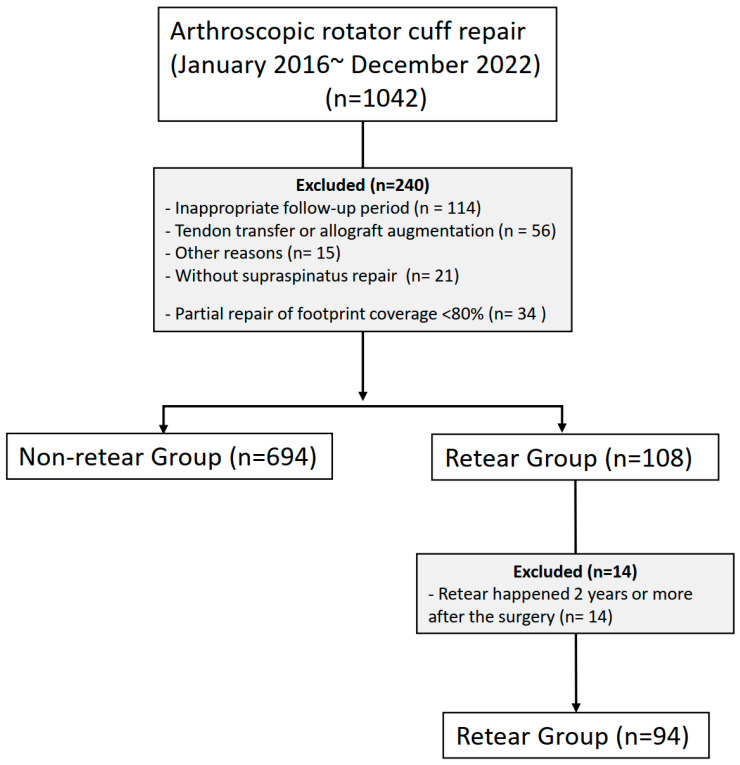
Flowchart of the number of included and excluded patients.

We excluded cases with additional allograft augmentation or tendon transfer procedures, neuromuscular diseases, a partial repair of footprint coverage of 80% or less, infections, or revision surgeries. Figure 1 shows the number of patients included in and excluded from this study.

Tendon integrity was assessed via magnetic resonance imaging (MRI) at 2 months and 1 year after the surgery and with sonography at 3 and 6 months after the surgery. An annual follow-up MRI at our institution was recommended after 1 year. For patients encountering financial difficulties, sonography was used instead of MRI. We defined a retear as occurring within 2 years after surgery, following the findings of Iannotti et al. [19] and Miller et al. [20] who reported that retears typically occur within the early postoperative period, from 3 months to 26 weeks after surgery.

### 2.1. Operative Technique and Rehabilitation

All surgical procedures were performed by a single senior surgeon (YSK) with the patient in the lateral decubitus position under general anesthesia. A capsulectomy was conducted if the patient demonstrated a limited preoperative range of motion (ROM) or findings of adhesive capsulitis intraoperatively. Various techniques were used for the repair of the torn tendon, including single- and double-row techniques. In some instances, the long head of the biceps tendon (LHBT) was used for the repair of the torn tendon, either by rerouting the biceps or using a biceps augmentation technique [21]. Subscapularis (SSC) repair, biceps tenotomy or tenodesis, and acromioplasty were performed as necessary.

Postoperatively, patients were immobilized for 4–6 weeks using an abduction brace. Passive ROM exercises commenced after the immobilization period of 4–6 weeks. Active ROM and muscle strengthening exercises began 8 weeks following surgery. For patients who underwent capsulectomy, passive ROM exercises were initiated 1 day after surgery, and active ROM and muscle strengthening exercises commenced 8 weeks after surgery.

### 2.2. Variable Selection and Clinical Assessment

All preoperatively measured variables that could be retrieved through electronic medical record (EMR) review were considered for inclusion in this study. Variables that were missing in >10% of patients were excluded from consideration.

The size of the SSP tear was measured following intraoperative debridement. Tear retraction was measured as the distance from the medial margin of the torn tendon to its original footprint. The anterior to posterior size of the tear was determined by measuring the longest distance from the anterior to the posterior margin of the torn tendon. The condition of the SSC muscle was assessed according to the Lafosse classification [22]. Fatty infiltrations of the SSP, ISP, and SSC muscles were evaluated on preoperative MRI (Verio, 3-T, Siemens, Munich, Germany) in accordance with the Goutallier classification [23]. Muscle atrophy of the SSP, ISP, and SSC was assessed using the tangent sign on preoperative MRI [24]. Biceps lesions were classified into five groups: 0: intact; 1: tendinopathy; 2: partial tear < 50%; 3: partial tear > 50%; and 4: complete tear. Retears were assessed by MRI or sonography (HD11xe Ultrasound System; Philips, Amsterdam, The Netherlands), with the integrity of the tendon evaluated with MRI using the classification proposed by Sugaya et al. [25], where types IV and V were categorized as retears. Sonography assessments were carried out by orthopedic clinical fellows, considering a hypoechoic or anechoic defect extending from the bursal to the articular surface as indicative of a retear.

For subjective variables, preoperatively recorded validated PROMs were used. These included the VAS for pain, which ranges from 0 to 10; the ASES score; the Constant–Murley (CM) score; and the Korean Shoulder Scoring System (KS) score. The preoperative active ROM, including forward flexion, external rotation in the side arm position, external rotation in the abduction position, and internal rotation, was quantified using a handheld goniometer. These measurements were consistently performed by a single senior surgeon, with internal rotation documented as the highest vertebral level the thumb could reach along the spine when extended upward. Demographic factors, such as age, sex, smoking status, body mass index (BMI), the presence of diabetes mellitus (DM), and whether the dominant or non-dominant arm was involved, were also included as feature variables in the model.

### 2.3. Model Development and Feature Importance

To predict retears, we trained five distinct machine learning models: Extreme Gradient Boosting (XGBoost), Random Forest (RF), Support Vector Machine (SVM), Neural Network (NN), and logistic regression (LR). These models were selected due to their demonstrated effectiveness in forecasting medical outcomes [11,12,17,18]. Logistic regression was used as a baseline for comparative analysis against the more advanced models, given its conventional use in risk prediction. For LR analysis, both univariate and multivariate analyses were conducted, yielding odds ratios (ORs) and *p*-values for each factor.

The dataset was split into training and test sets at an 8:2 ratio. The dataset was initially imbalanced with a retear rate of 11.9%, so it was balanced using the synthetic minority oversampling technique (SMOTE), applied solely to the training set [26,27]. To ensure transparency and confirm that the SMOTE did not substantially alter the original data distributions, we have included Supplementary Materials presenting pairplots comparing variable distributions before and after applying the SMOTE. Within the training set, three-fold cross-validation was performed, along with a grid search to fine-tune the hyperparameters of the models. The integrity of the variable distributions post oversampling was verified to ensure there was no distortion.

Standardization was applied to all continuous variables to normalize scale variability, while nonordinal categorical variables were converted into dummy variables. The performance of the final models was evaluated using data from the test set, with performance metrics including the area under the receiver operating characteristic curve (AUC), predictive accuracy, sensitivity, and specificity. The receiver operating characteristic (ROC) curve was plotted to compute the AUC and Youden’s J Index [28].

To assess variable importance in the XGBoost model, SHAP values were calculated, as outlined in previous research [15,29]. In the RF model, an intrinsic feature importance metric was used for ranking features [16]. Our machine learning models were developed using Python Version 3.9.4 and the open-source library scikit-learn 1.1.0.

### 2.4. Statistical Analysis

Baseline characteristics for patients with and without retears are presented as the mean and standard deviation (SD) for continuous variables and counts for categorical variables. To determine differences between the two groups, continuous variables were analyzed using the *t*-test, and categorical variables were analyzed with Fisher’s exact test. The outcomes from the univariate and multivariate LR analyses are reported as ORs with 95% confidence intervals (CIs). A *p*-value of less than 0.05 was considered statistically significant. Statistical analyses were conducted using R version 4.2.1.

## 3. Results

### 3.1. Demographic Features

The final dataset included 788 patients, categorized into non-retear and retear groups. Table 1 provides a detailed distribution of demographic features. Retears were observed in 11.9% of cases (94/788). The average age was significantly higher in the retear group (*p* < 0.01). The tear profile of the retear group demonstrated a higher incidence of full-thickness tears and larger tear sizes in both the anterior–posterior (AP) and medial–lateral (ML) dimensions compared to the non-retear group (*p* < 0.05). There were significant differences in the FI proportions between the two groups, although the majority of Goutallier stages for all three muscles in both groups were 0 and I. No significant differences were observed in the muscle atrophy status between the groups. Similarly, clinical outcomes, including ROM and clinical scores, showed no significant differences.

### 3.2. Performance of Machine Learning Algorithms

**Figure 2:**
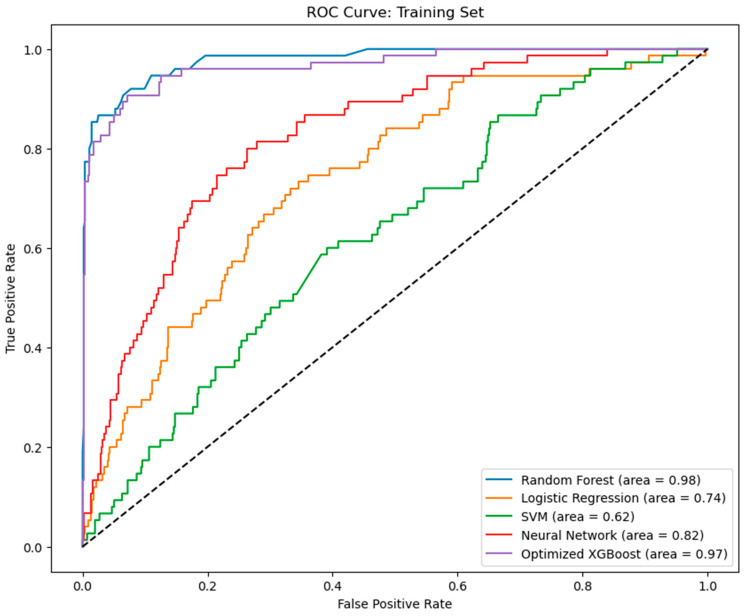
Receiver operating characteristic (ROC) curve of the three-fold cross-validation results.

The performance of the five machine learning algorithm models within the training and validation cohorts, based on the AUC through three-fold cross-validation, is depicted in Figure 2. The AUC for the RF model was 0.9790 (SD = 0.0051); for the LR model, it was 0.8974 (SD = 0.0235); for the SVM model, it was 0.6379 (SD = 0.0145); for the NN model, it was 0.9354 (SD = 0.0037); and for the XGBoost model, it was 0.9785 (SD = 0.0011). Details on the performance of the models on the independent test set are provided in Table 2. The XGBoost and RF algorithms were the top-performing models across both the cross-validation process and the test set evaluation.

### 3.3. Feature Importance in the XGBoost and Random Forest Model

**Figure 3:**
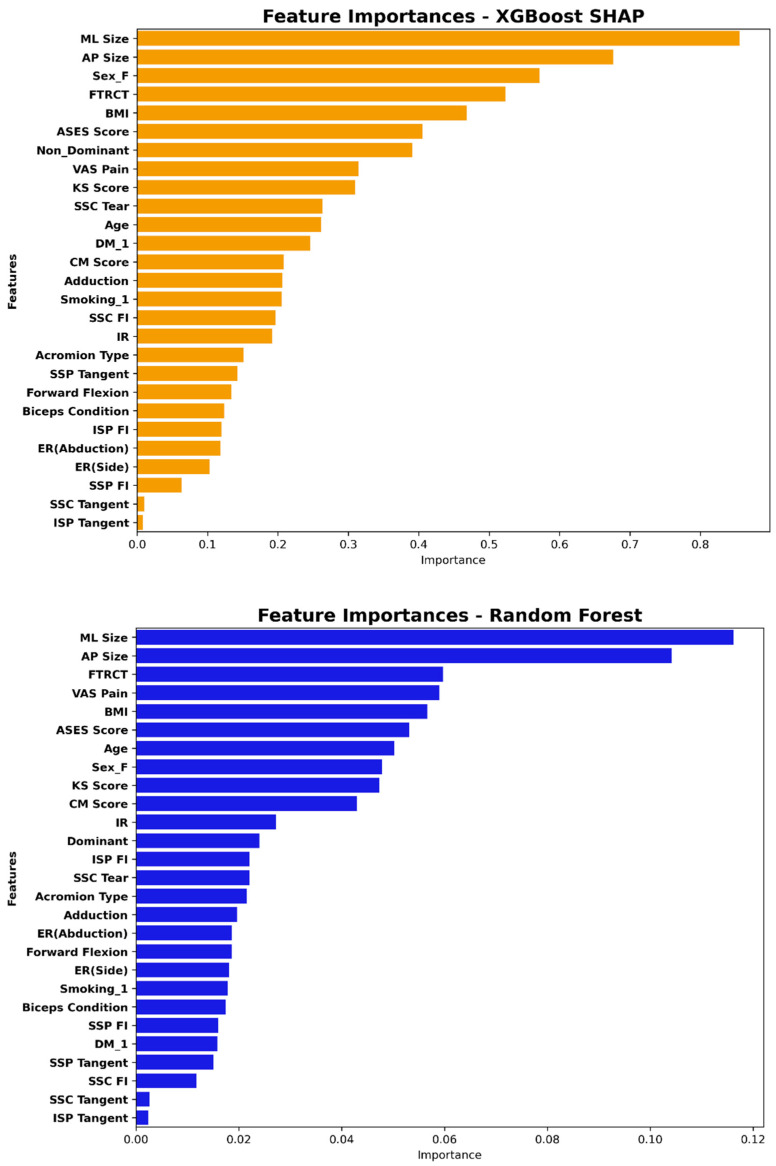
Feature importance derived from the XGBoost-SHAP and Random Forest (RF) models. SHAP (SHapley Additive exPlanation) values quantify the contribution of each feature to the model’s prediction by measuring how each feature affects the prediction when interacting with other features. RF importance scores reflect the significance of each feature based on how much it reduces impurity across decision trees. BMI, body mass index; DM, diabetes mellitus; AP, anterior–posterior; ML, medial–lateral; FI, fatty infiltration; abd., abduction; VAS, visual analogue scale; FTRCT, full-thickness rotator cuff tear; ASES, American Shoulder and Elbow Surgeon; CM, Constant–Murley; KS, Korean Shoulder.

Figure 3 illustrates the feature importance plots derived from the XGBoost and RF models, which were the top-performing models. The five most important features identified by XGBoost included the tear size in the ML and AP dimensions, female sex, full-thickness tears, and BMI. The RF model showed similar feature importances to XGBoost but included the VAS pain score instead of female sex. The tear size in the ML dimension was the most predictive feature for the presence of a retear in both models, with the AP dimension tear size as the next most important factor. Full-thickness tears and BMI were also among the top five most important factors in both analyses.

### 3.4. Univariate and Multivariate Logistic Regression Analysis

Univariate LR analysis identified age, the presence of a full-thickness tear, the tear size in both the AP and ML dimensions, the condition of the biceps tendon, the FI of the SSP, SSC, and ISP muscles, and SSP atrophy as variables significantly associated with retear after ARCR (*p* < 0.05). However, multivariate analysis demonstrated that only age (OR = 1.040, 95%CI [1.006–1.075], *p* = 0.02) and the ML tear size (OR = 1.580, 95%CI [1.052–2.392], *p* = 0.029) were significantly related to the occurrence of a retear. The detailed results of both univariate and multivariate analyses are provided in Table 3.

## 4. Discussion

We developed and validated multiple widely used machine learning algorithms to predict the occurrence of retears after ARCR. Among five machine learning models, the RF and XGBoost models were the top-performing models, surpassing the classical LR model. The size of the tear in both the AP and ML dimensions, BMI, full-thickness tears, female sex, and VAS pain scores were identified as significant in terms of feature importance for the prediction of retear occurrence. The most influential features were remarkably similar across both models. Traditional multivariate LR analysis, however, highlighted significant ORs for age and tear size in the medial-to-lateral dimension only.

The top five risk factors identified by the two highest-performing models closely align with findings from previous studies. For instance, Manop et al. [6] conducted a multivariate LR analysis on risk factors for ARCR failure and identified the AP tear size, retraction, BMI, and high work activity as significant predictors. The dimension of the tear, whether ML or AP, has been frequently cited as a primary factor in retear occurrence [2,3,6,7]. In addition, a high BMI has been extensively associated with poorer healing outcomes after ARCR, as evidenced by several studies, including meta-analyses [7,30,31]. Our findings, which highlight the tear size, full-thickness tears, BMI, female sex, and the VAS pain score as influential risk factors, are in partial agreement with previous studies [6,7]. In particular, BMI and female sex are supported by recent studies, which have similarly identified these factors as influential in rotator cuff healing, thereby reinforcing the clinical relevance of our model [32,33,34].

One novel aspect of our study was the concurrent comparison of subjective and objective factors. The results demonstrated that the feature importances of subjective factors were not included in the top five ranking except for the VAS pain score, which ranked fourth in the RF model. PROM values face challenges in being included in risk factor surveys due to reasons such as their highly subjective nature or low response rates [10]. However, PROMs should also be considered valuable measurements to evaluate prognosis because they are directly related to the patient’s subjective satisfaction. Although only the RF model highlighted VAS pain as a highly important factor, high levels of preoperative pain may contribute to poorer retear outcomes, potentially explaining the lower postoperative PROM results documented in other research [8,9,10].

Recognizing high-risk factors in clinical practice could help surgeons better identify patients who might be at increased risk. For instance, patients with larger tear sizes, patients with an elevated BMI, patients with higher preoperative pain scores, or female patients may benefit from targeted preoperative counseling regarding their heightened retear risk. These patients could also receive individualized postoperative management strategies, such as more rigorous follow-up imaging or tailored rehabilitation protocols. Thus, the integration of these insights into clinical practice could foster a more personalized approach to patient care and potentially improve clinical outcomes. Future studies should aim to develop and validate practical tools, such as individualized risk prediction models, to further enhance clinical decision-making.

Our study did not prioritize frequently suggested risk factors for rotator cuff healing, such as FI, DM, or smoking. This highlights the complexity of risk factor analysis and the impact of methodological approaches on outcomes. While FI was significant in univariate analysis, machine learning revealed a different hierarchy of importance. Real-world scenarios involve multifactorial interactions where dominant factors may overshadow others in predictive models. Therefore, our findings advocate for a multifactorial and methodologically diverse approach to understanding retear risk after ARCR. Traditional risk factor analysis in orthopedic research has relied on univariate and multivariate LR models, which may not fully capture complex relationships.

The machine learning models identify BMI and female sex as key predictors, but the multivariate logistic regression only finds age and the ML tear size to be significant. The apparent discrepancy arises because machine learning algorithms can detect nonlinear interactions and subtle effects, which may not be identified by traditional multivariate logistic regression. Thus, while age and the ML tear size were statistically significant in the logistic model, BMI and female sex may still exert clinically meaningful influences captured by the machine learning approach. Our study used various machine learning algorithms for a more complex understanding. SHAP values and RF feature importance have provided transparent insights into the decision-making processes of predictive models [11,12,17,18]. This methodological shift aligns with the growing call for advanced analytical techniques in medical research, as highlighted by Beam et al. [35] in their discussion on the potential of machine learning in healthcare. Similar to findings by Rajkomar et al. [36], our study demonstrates that machine learning algorithms can surpass conventional statistical methods in various medical fields by enhancing predictive accuracy and providing a deeper understanding of risk factor dynamics.

Our study has some limitations. First, all procedures were conducted by a single senior surgeon with over two decades of experience, using a variety of repair techniques. This may limit the generalizability of the results. Future studies could use our risk factors to assess the outcomes from multiple surgeons across different centers. Second, not all measurable features were included, such as the critical shoulder angle or acromiohumeral distance, which could have influenced our findings. Further studies should incorporate these parameters to provide a more comprehensive assessment of risk factors. Additionally, retears were assessed using MRI or ultrasound at different postoperative intervals, potentially introducing variability into the accuracy or consistency of retear diagnosis. Also, despite high cross-validation AUCs (~0.98), the drop to ~0.67 on the test set likely reflects the inherent challenge of overfitting in complex models applied to retrospective data. This limitation is common in studies with imbalanced outcomes, and our primary goal was to explore risk factor importance rather than achieve perfect predictive accuracy. Another limitation is the lack of an external validation set. Although our study relied on three-fold cross-validation—an approach commonly used in similar retrospective studies—future research should consider external validation or bootstrapping to further verify these findings. Our study focused exclusively on preoperative risk factors and did not account for postoperative rehabilitation adherence, which could influence retear rates. Finally, the retrospective nature of this review may introduce certain inaccuracies, although the medical records were carefully examined and considered reliable. While our study provides valuable insights, the use of a single-center, retrospective dataset may limit generalizability and introduce bias, a limitation inherent to many such studies.

## 5. Conclusions

Machine learning algorithms can predict retears with greater accuracy than conventional LR. Through machine learning models, the tear size and BMI were identified as the most influential risk factors, while the VAS pain score and female sex should also be considered risk factors for retears after ARCR.

## Figures and Tables

**Table 1:** Patient population (mean ± SD or n).

	Non-Retear (n = 694)	Retear (n = 94)	*p*-Value
Sex, male–female	253:441	64:30	0.424
Age, years	61.7 ± 8.4	64.9 ± 7.4	0.001
BMI,	24.6 ± 3.2	24.1 ± 2.9	0.115
DM, yes–no	95:599	17:77	0.270
Smoking, yes–no	54:640	11:83	0.228
Dominant side, yes–no	488:206	72:22	0.227
Acromion Type, 1:2:3	94:511:89	16:61:17	0.055
Full-thickness tear, +:−	442:252	84:10	<0.001
AP tear size, cm	1.2 ± 0.7	1.7 ± 0.9	<0.001
ML tear size, cm	1.3 ± 0.9	1.9 ± 1.0	<0.001
Subscapularis tear, I:II:III:IV:V	440:133:91:21:9	55:26:9:3:1	0.454
Biceps condition, 0:1:2:3:4	341:152:91:101:9	36:17:17:24:0	0.029
Supraspinatus FI, 0:I:II:III:IV	137:373:134:37:13	10:42:29:12:1	0.002
Subscapularis FI, 0:I:II:III:IV	452:230:12:0:0	47:44:2:0:1	0.004
Infraspinatus FI, 0:I:II:III:IV	375:248:63:7:1	36:46:11:0:1	0.015
Supraspinatus Tangent, 1:0	511:183	35:59	0.070
Subscapularis Tangent, 1:0	676:18	2:92	1.000
Infraspinatus Tangent, 1:0	680:14	3:91	0.493
Forward flexion	140.9 ± 15.3	142.8 ± 12.8	0.189
External rotation (side)	82.3 ± 13.3	83.4 ± 12.6	0.411
External rotation (abd.)	84.0 ± 12.7	84.8 ± 10.7	0.473
Internal rotation	9.2 ± 3.4	9.7 ± 3.2	0.157
Adduction	23.3 ± 4.8	24.0 ± 4.4	0.179
VAS for pain	4.2 ± 2.0	3.8 ± 2.4	0.206
ASES score	57.8 ± 18.9	58.7 ± 22.4	0.710
CM score	69.1 ± 16.5	68.8 ± 16.1	0.873
KS score	65.9 ± 15.9	64.3 ± 16.1	0.368

BMI, body mass index; DM, diabetes mellitus; AP, anterior–posterior; ML, medial–lateral; FI, fatty infiltration; abd., abduction; VAS, visual analogue scale; ASES, American Shoulder and Elbow Surgeon; CM, Constant–Murley; KS, Korean Shoulder.

**Table 2:** Model performances using test set in five models.

Model	AUC-ROC	Accuracy	Sensitivity	Specificity
RF	0.653	0.576	0.737	0.554
LR	0.570	0.430	0.842	0.374
SVM	0.597	0.380	0.947	0.302
NN	0.641	0.487	0.895	0.432
XGBoost	0.676	0.506	0.842	0.460

**Table 3:** Univariate and multivariate logistic regression analysis for retears.

Variables	Univariate Analysis		Multivariate Analysis	
	OR (95%CI)	*p*-Value	OR (95%CI)	*p*-Value
Sex (M)	0.817 (0.510–1.284)	0.39	0.614 (0.330–1.099)	0.11
Age	1.051 (1.023–1.081)	<0.001	1.040 (1.006–1.075)	0.02
BMI	0.947 (0.879–1.016)	0.140	0.958 (0.883–1.035)	0.289
DM	1.392 (0.767–2.405)	0.254	1.240 (0.649–2.274)	0.5
Smoking	1.571 (0.753–3.017)	0.198	2.192 (0.886–5.231)	0.081
Dominant side	1.382 (0.847–2.335)	0.209	1.318 (0.759–2.361)	0.339
Acromion Type				
Type 1	-[Reference]		-[Reference]	
Type 2	0.701 (0.396–1.306)	0.241	0.514 (0.268–1.022)	0.05
Type 3	1.122 (0.533–2.373)	0.761	0.673 (0.287–1.569)	0.357
FTRCT	4.789 (2.559–9.985)	<0.001	2.106 (0.972–4.898)	0.069
AP size	2.345 (1.794–3.081)	<0.001	1.476 (0.926–2.348)	0.1
ML size	2.122 (1.690–2.678)	<0.001	1.580 (1.052–2.392)	0.029
SSC Lafosse	1.008 (0.790–1.260)	0.945	0.920 (0.700–1.185)	0.532
BC	1.265 (1.060–1.506)	0.009	1.148 (0.938–1.400)	0.176
SSC FI	1.787 (1.234–2.579)	0.002	1.258 (0.714–2.210)	0.425
SSP FI	1.487 (1.181–1.868)	<0.001	0.996 (0.645–1.544)	0.985
ISP FI	1.406 (1.060–1.805)	0.016	0.864 (0.508–1.454)	0.585
SSC Tangent	0.816 (0.128–2.891)	0.788	0.425 (0.520–2.194)	0.354
SSP Tangent	1.656 (1.047–2.588)	0.028	0.683 (0.363–1.257)	0.227
ISP Tangent	1.601 (0.364–5.021)	0.466	1.127 (0.193–5.450)	0.885
Forward flexion	1.010 (0.994–1.029)	0.25	1.008 (0.974–1.046)	0.673
ER (side)	1.007 (0.991–1.028)	0.431	1.008 (0.959–1.054)	0.748
ER (abduction)	1.006 (0.989–1.028)	0.528	0.980 (0.946–1.032)	0.347
IR	1.048 (0.981–1.128)	0.177	1.022 (0.919–1.142)	0.701
Adduction	1.031 (0.984–1.082)	0.205	1.019 (0.955–1.077)	0.491
VAS pain	0.925 (0.830–1.028)	0.152	0.849 (0.668–1.080)	0.181
ASES score	1.002 (0.991–1.014)	0.672	0.986 (0.956–1.016)	0.374
CM score	0.999 (0.986–1.012)	0.873	1.032 (0.998–1.069)	0.073
KS score	0.994 (0.981–1.007)	0.361	0.972 (0.967–1.008)	0.125

BMI, body mass index; DM, diabetes mellitus; AP, anterior–posterior; ML, medial–lateral; FI, fatty infiltration; abd., abduction; VAS, visual analogue scale; FTRCT, full-thickness rotator cuff tear; ASES, American Shoulder and Elbow Surgeon; CM, Constant–Murley; KS, Korean Shoulder; SSP, supraspinatus; SSC, subscapularis; ISP, infraspinatus.

## Data Availability

The datasets used and/or analyzed during the current study are available from the corresponding author on reasonable request.

## Supplementary Materials

The following supporting information can be downloaded at https://www.mdpi.com/article/10.3390/jcm14061843/s1. S1.1. Pairplot displaying the distribution and relationships among variables in the original dataset; S1.2 Pairplot displaying the distribution and relationships among variables in the SMOTE-balanced dataset.
