# Supplementary material for: Predicting Major Preoperative Risk Factors for Retears After Arthroscopic Rotator Cuff Repair Using Machine Learning Algorithms
Source: J Clin Med. 2025 Mar 9;14(6):1843. doi: 10.3390/jcm14061843 (PMC11943030; doi:10.3390/jcm14061843)

[Supplementary figures]

S1.1. Pairplot displaying the distribution and relationships among variables in the original dataset.

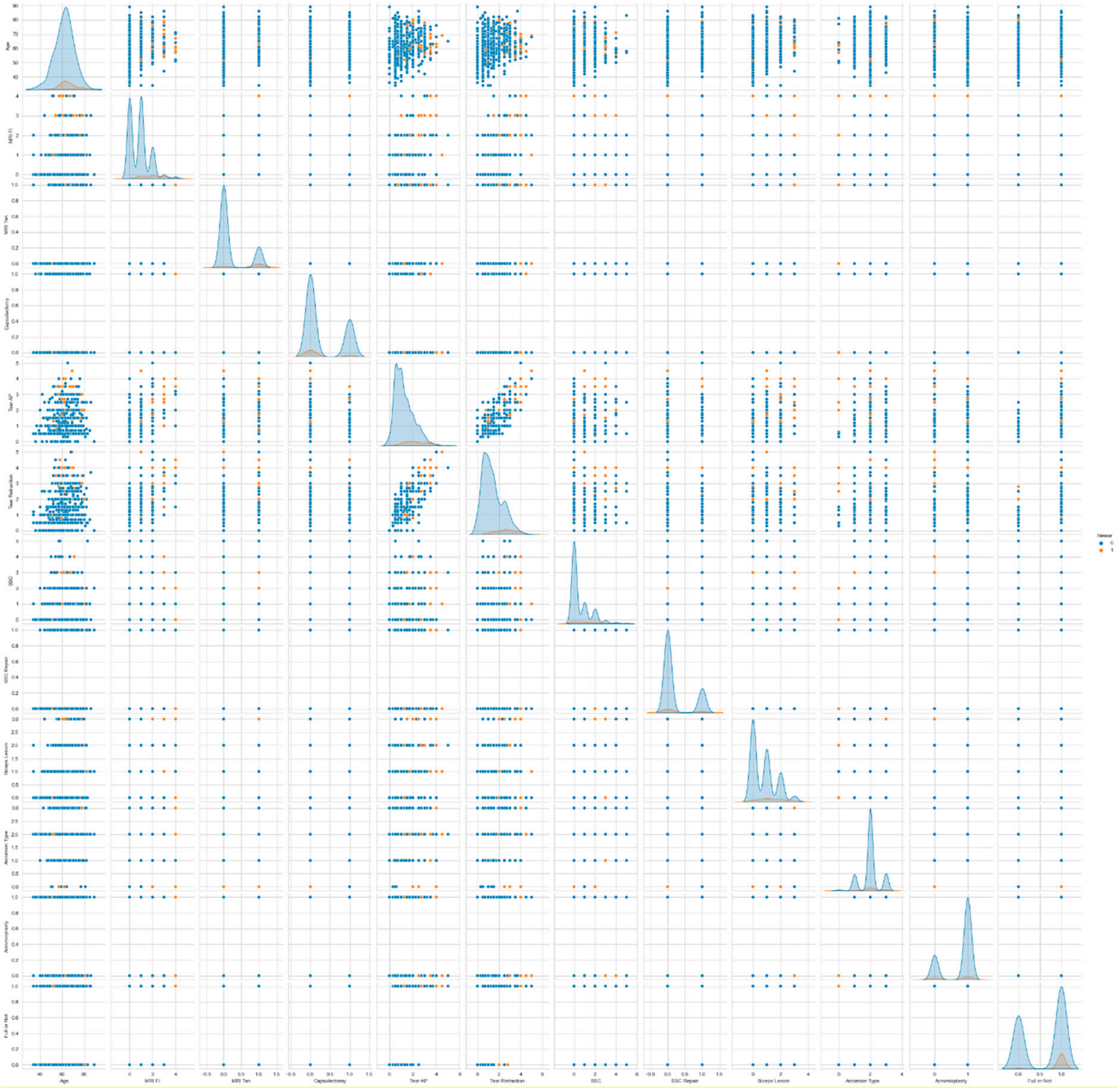

S1.2 Pairplot displaying the distribution and relationships among variables in the SMOTE-balanced dataset.

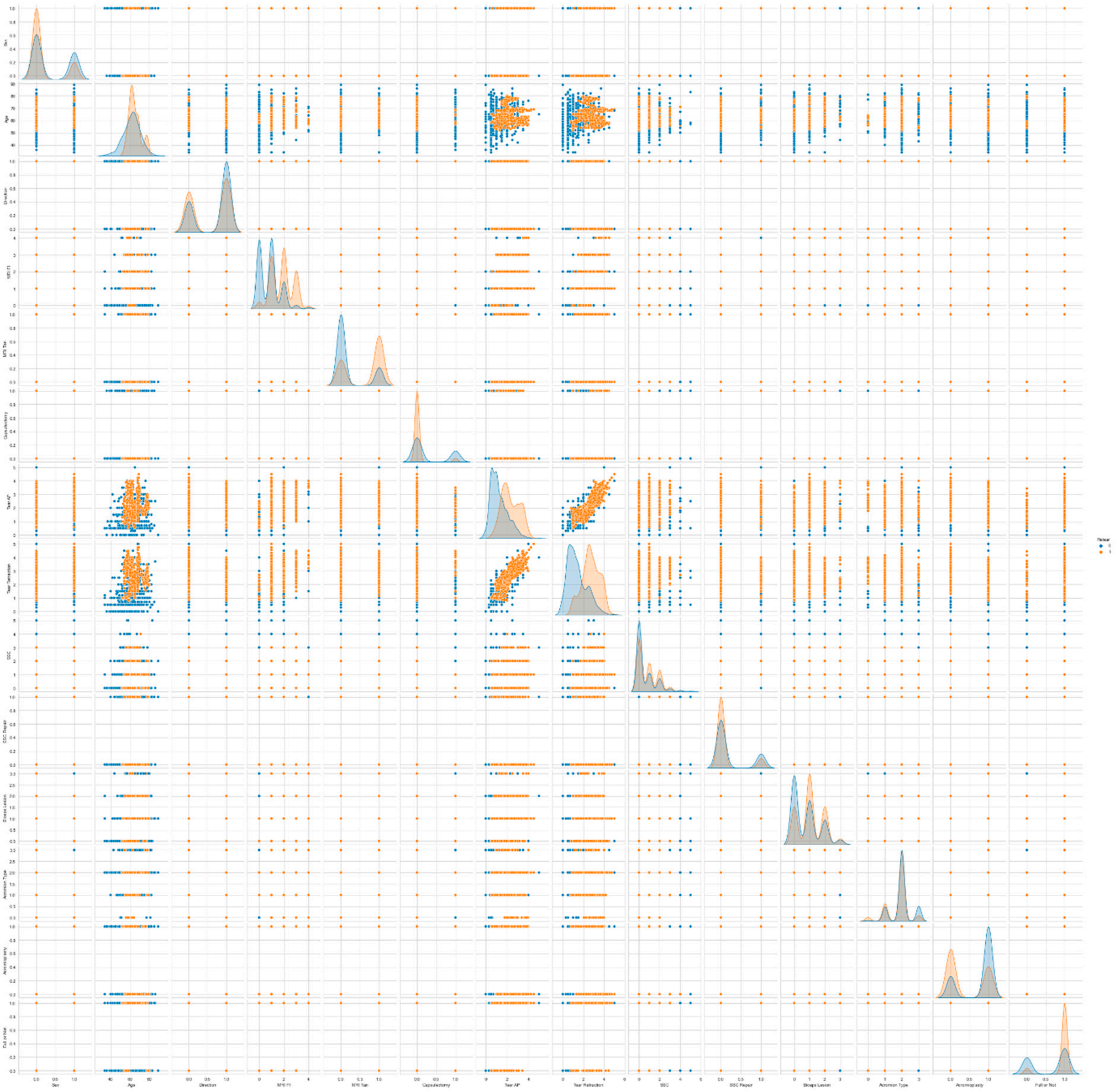

Supplement: Supplementary file 1 [file jcm-14-01843-s001.zip › Supplementary figures.pdf]
