# Supplementary figures and images for: Predicting Major Preoperative Risk Factors for Retears After Arthroscopic Rotator Cuff Repair Using Machine Learning Algorithms
Source: J Clin Med. 2025 Mar 9;14(6):1843. doi: 10.3390/jcm14061843 (PMC11943030; doi:10.3390/jcm14061843)

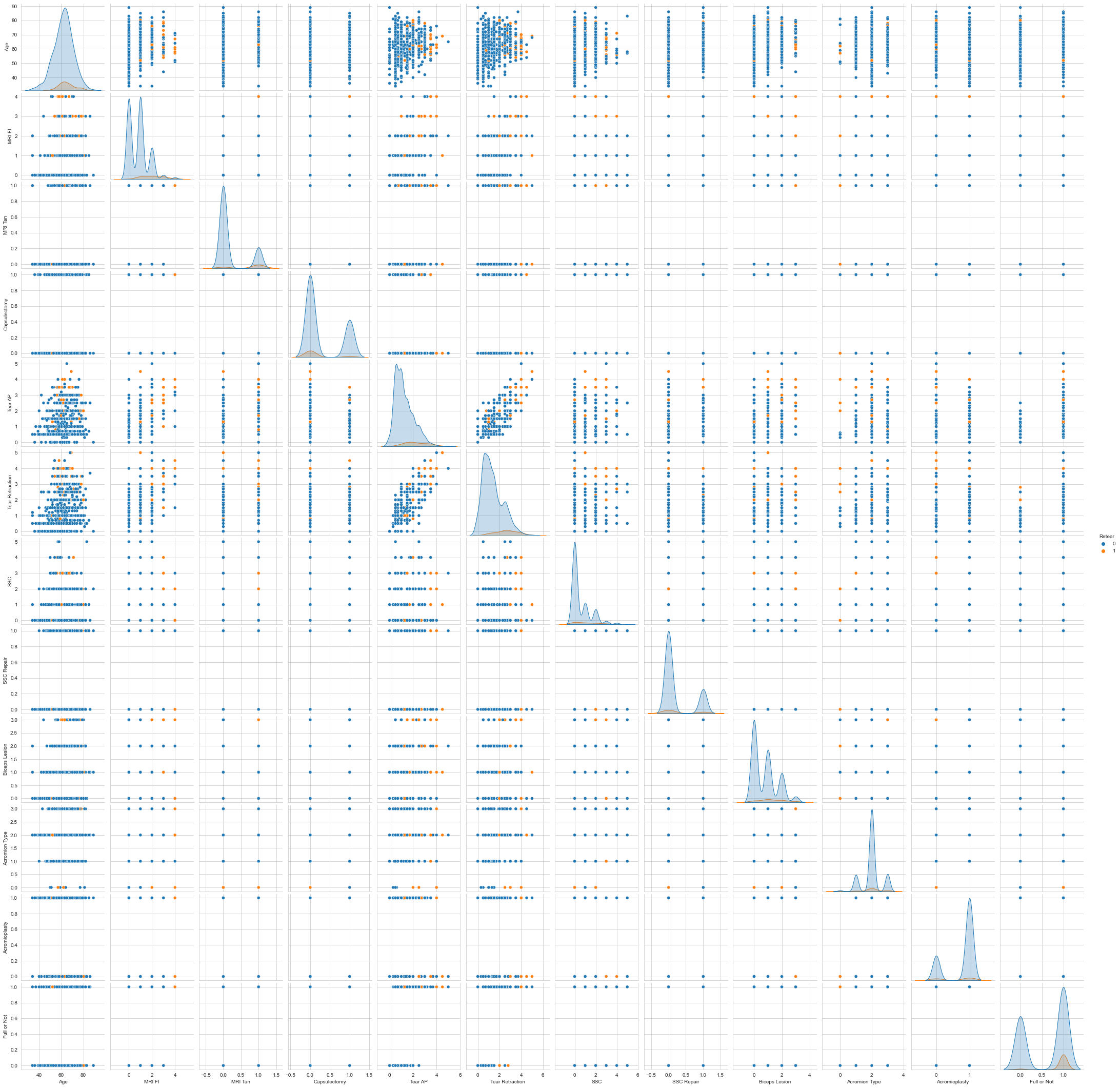

Supplement: Supplementary file 1 [file jcm-14-01843-s001.zip › PairPlot.png]

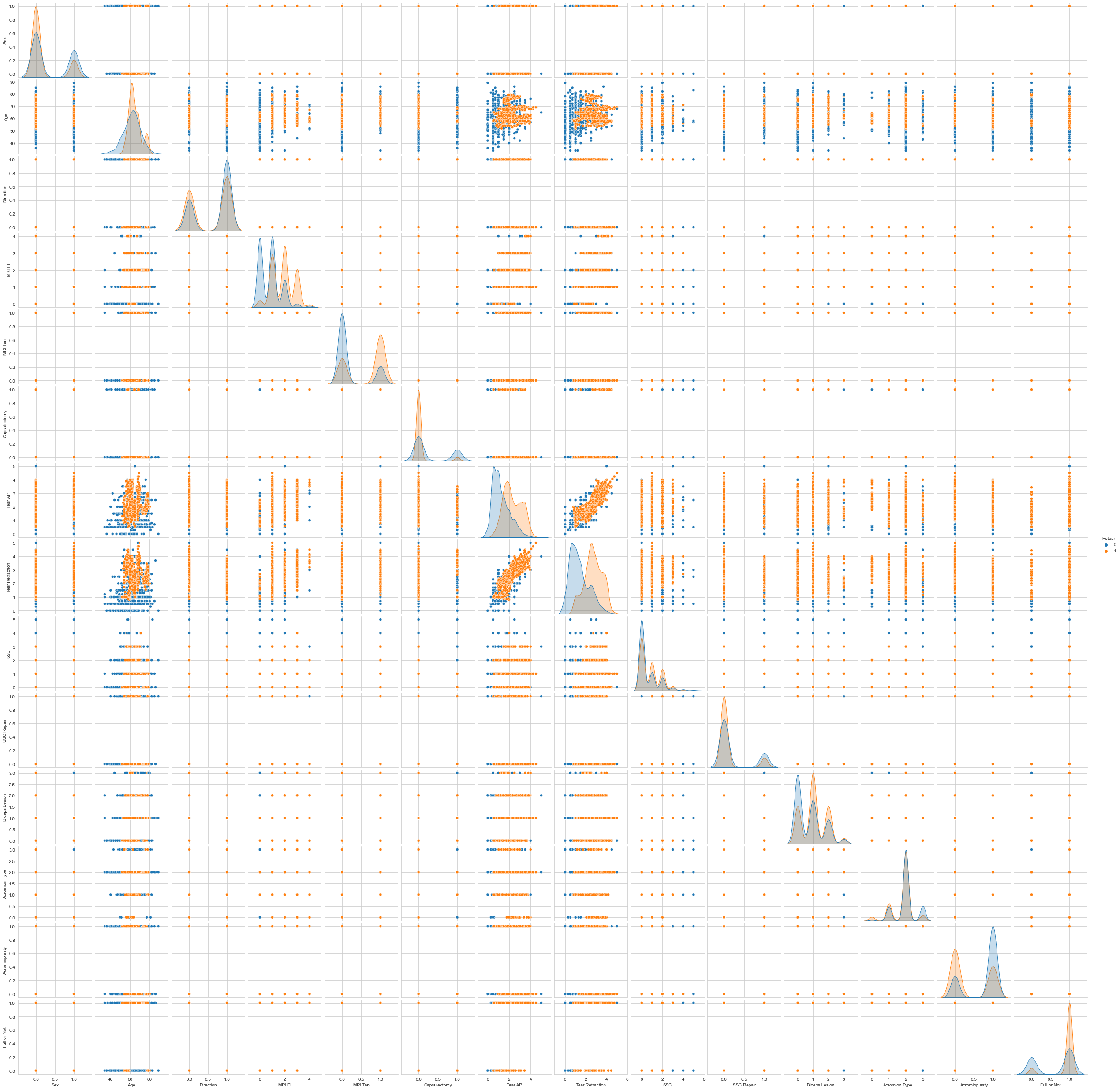

Supplement: Supplementary file 1 [file jcm-14-01843-s001.zip › PairPlot_SMOTE.png]
